# Cracking the laugh code: laughter through the lens of biology, psychology and neuroscience

**DOI:** 10.1098/rstb.2022.0159

**Published:** 2022-11-07

**Authors:** Fausto Caruana, Elisabetta Palagi, Frans B. M. de Waal

**Affiliations:** ^1^ Institute of Neuroscience, National Research Council of Italy (CNR), Via Volturno 39/E, 43125 Parma, Italy; ^2^ Unit of Ethology, Department of Biology, University of Pisa, via A. Volta 6, 56126, Pisa, Italy; ^3^ Emory University, Atlanta, GA 30322, USA

## 1. Introduction

Laughter affects conversational schemes, supports speech production, establishes social bonds and is connected to playfulness. Despite the pervasiveness of this behaviour, research on laughter has long been underdeveloped, probably because it does not sound like a serious topic. Also, its social and expressive nature represents a major technical difficulty for both laboratory and naturalistic studies. The quest to uncover the processes underlying the production and perception of laughter is still in its early days, but a renewed interest in this behaviour has boosted the emergence of new ecological studies in a set of fields encompassing a broad spectrum of disciplines.

This Theme Issue aims to tackle the biological, psychological, neural and cultural underpinnings of laughter in humans and other animals from a naturalistic and evolutionary perspective. A new naturalistic account of laughter has been boosted by the work of the psychologist Robert Provine (1943–2019) and the neuroscientist Jaak Panksepp (1943–2017), to whom this issue is dedicated. According to this view, laughter must be studied considering the behavioural intentions it conveys and the response it elicits in the recipient. Notably, such an approach shifts the focus of attention from the cognitive underpinnings of humour processing to the adaptive socio-emotional nature of laughter, which signals not only reward and amusement but also affiliation and benign intentions.

The naturalistic study of human laughter is also supported by the wide acceptance that other species show a homologue of laughter. Playful laugh expressions of the great apes and other primates not only morphologically resemble human laughter but they share important social functions and neural substrates. However, similar play vocalizations have probably evolved multiple times in evolution, as in the case of play vocalizations in rats.

This Theme Issue synthesizes existing knowledge on laughter in humans and other animals through the lens of an evolutionary approach. The great variety of contributions proposed in this issue includes new insights from ethology, anthropology, social psychology and cognitive and affective neuroscience, thus providing an overarching perspective of the laughter phenomenon.

The issue consists of two main sections:

(i) A first section is theoretical in nature. An opinion piece provides a unifying framework across the different disciplines also suggesting further ideas to expand the knowledge through a comparative approach (Palagi *et al*. [1]). Then four reviews frame laughter studies in the fields of anthropology (Dunbar [2]), ethology (Davila-Ross & Palagi [3]), psychology (Scott *et al*. [4]) and cross-cultural studies (Bryant & Bainbridge [5]).
(ii) A second section is entirely dedicated to new empirical data, with studies tackling the issue of laughter in the fields of behavioural studies (Burke *et al*. [6], Proelss *et al*. [7]), social psychology (Hess [8], Wood [9]), consciousness studies (Prochazkova *et al*. [10]), neuroimaging (Wattendorff *et al*. [11], Belyk *et al*. [12]), and computational (Celeghin *et al*. [13]), clinical (Sessa *et al*. [14]) and system neuroscience (Zauli [15], Sun [16]).

Our hope is that the present Theme Issue will contribute not only to conciliate the different visions of laughter emerging by the different disciplinary origins of thinkers, but also to provide new data supporting this new unifying naturalistic vision.

## Data accessibility

This article has no additional data.

## Authors' contributions

F.C.: writing—original draft, writing—review and editing; E.P.: writing—original draft, writing—review and editing; F.B.M.d.W.: writing—original draft, writing—review and editing.

All authors gave final approval for publication and agreed to be held accountable for the work performed therein.

## Conflict of interest declaration

This theme issue was put together by the Guest Editor team under supervision from the journal's Editorial staff, following the Royal Society's ethical codes and best-practice guidelines. The Guest Editor team invited contributions and handled the review process. Individual Guest Editors were not involved in assessing papers where they had a personal, professional or financial conflict of interest with the authors or the research described. Independent reviewers assessed all papers. Invitation to contribute did not guarantee inclusion.

## Funding

We received no funding for this study.
